# Maine Veterinary Medical Association

**Published:** 1900-01

**Authors:** I. L. Salley

**Affiliations:** Secretary


Secretary.

### MAINE VETERINARY MEDICAL ASSOCIATION.

THE regular meeting was held at Waterville, October 13, 1899.

At 10 o'clock a clinic was begun, which lasted all day, with the following operations:

Fistula of the inferior maxilla, Dr. West; cauterization of spavin, Drs. Freeman, Purcell, and Blakely; removal of tumors, Dr. Joly; fistula of the withers, Dr. West; castration of ridgling, Dr. Salley.

All the operations except cauterization of spavin were performed under the influence of chloroform.

All were well pleased with the day's operations, and felt well repaid for the time and trouble to attend the meeting.

At 7.30 P.M. a meeting was held at the Elmwood, President West in the chair. Drs. Freeman, Joly, Purcell, Blakely, West, and Salley responded to the roll-call. Minutes of previous meetings were read and adopted. Voted to adjourn to meet at Augusta in January.

DR. I. L. SALLEY,
Secretary.

---

Dr. William J. Denton, of Philadelphia, is the only breeder of the Pekinese Spaniels in America and a historian on the Chinese development of these curios in the canine world.

